# S-Gene Target Failure as an Effective Tool for Tracking the Emergence of Dominant SARS-CoV-2 Variants in Switzerland and Liechtenstein, Including Alpha, Delta, and Omicron BA.1, BA.2, and BA.4/BA.5

**DOI:** 10.3390/microorganisms12020321

**Published:** 2024-02-03

**Authors:** Dominique Hilti, Faina Wehrli, Sabine Berchtold, Susanna Bigler, Thomas Bodmer, Helena M. B. Seth-Smith, Tim Roloff, Philipp Kohler, Christian R. Kahlert, Laurent Kaiser, Adrian Egli, Lorenz Risch, Martin Risch, Nadia Wohlwend

**Affiliations:** 1 Laboratory Dr. Risch, 9470 Buchs, Switzerlandlorenz.risch@risch.ch (L.R.); nadia.wohlwend@risch.ch (N.W.); 2 Institute of Laboratory Medicine, Private University in the Principality of Liechtenstein (UFL), 9495 Triesen, Liechtenstein; 3 Institute of Medical Microbiology, University of Zurich, 8006 Zurich, Switzerland; 4 Zentrallabor, Kantonsspital Graubünden, 7000 Chur, Switzerland; 5 Division of Infectious Diseases and Hospital Epidemiology, Cantonal Hospital St. Gallen, 9007 St. Gallen, Switzerland; 6 Division of Infectious Diseases, Geneva University Hospitals, 1205 Geneva, Switzerland; 7 Geneva Centre for Emerging Viral Diseases, Geneva University Hospitals, 1205 Geneva, Switzerland

**Keywords:** S-gene target failure, whole-genome sequencing, SARS-CoV-2, surveillance

## Abstract

During the SARS-CoV-2 pandemic, the Dr. Risch medical group employed the multiplex TaqPath^TM^ COVID-19 CE-IVD RT-PCR Kit for large-scale routine diagnostic testing in Switzerland and the principality of Liechtenstein. The TaqPath Kit is a widely used multiplex assay targeting three genes (i.e., ORF1AB, N, S). With emergence of the B.1.1.7 (Alpha) variant, a diagnostic flaw became apparent as the amplification of the S-gene target was absent in these samples due to a deletion (ΔH69/V70) in the Alpha variant genome. This S-gene target failure (SGTF) was the earliest indication of a new variant emerging and was also observed in subsequent variants such as Omicron BA.1 and BA4/BA.5. The Delta variant and Omicron BA.2 did not present with SGTF. From September 2020 to November 2022, we investigated the applicability of the SGTF as a surrogate marker for emerging variants such as B.1.1.7, B.1.617.2 (Delta), and Omicron BA.1, BA.2, and BA.4/BA.5 in samples with cycle threshold (Ct) values < 30. Next to true SGTF-positive and SGTF-negative samples, there were also samples presenting with delayed-type S-gene amplification (higher Ct value for S-gene than ORF1ab gene). Among these, a difference of 3.8 Ct values between the S- and ORF1ab genes was found to best distinguish between “true” SGTF and the cycle threshold variability of the assay. Samples above the cutoff were subsequently termed partial SGTF (pSGTF). Variant confirmation was performed by whole-genome sequencing (Oxford Nanopore Technology, Oxford, UK) or mutation-specific PCR (TIB MOLBIOL). In total, 17,724 (7.4%) samples among 240,896 positives were variant-confirmed, resulting in an overall sensitivity and specificity of 93.2% [92.7%, 93.7%] and 99.3% [99.2%, 99.5%], respectively. Sensitivity was increased to 98.2% [97.9% to 98.4%] and specificity lowered to 98.9% [98.6% to 99.1%] when samples with pSGTF were included. Furthermore, weekly logistic growth rates (α) and sigmoid’s midpoint (t_0_) were calculated based on SGTF data and did not significantly differ from calculations based on comprehensive data from GISAID. The SGTF therefore allowed for a valid real-time estimate for the introduction of all dominant variants in Switzerland and Liechtenstein.

## 1. Introduction

Severe Acute Respiratory Syndrome Coronavirus 2 (SARS-CoV-2), first detected in Wuhan, China, in Winter 2019, was identified as the causative agent for the COVID-19 pandemic and is responsible for more than 773 million confirmed infections and close to 7 million deaths worldwide, as of December 2023 [1]. The first isolation of the novel agent, which took place on 7 January 2020, and sharing of its genetic sequence with the public by the Chinese authorities [2] led to the rapid development of numerous specific PCR-based diagnostic assays.

With respect to the scientific nomenclature, the World Health Organization (WHO) has developed criteria to classify SARS-CoV-2 variants based on their potential for increased transmissibility, virulence, clinical disease presentation, and the effectiveness of public health measures or available diagnostics, vaccines, and therapeutics [3]. The WHO uses a Greek nomenclature and differentiates between Variants Under Monitoring (VUMs), Variants of Interest (VOIs), and Variants of Concern (VOCs).

On 14 December 2020, the United Kingdom reported the first SARS-CoV-2 VOC, the Alpha variant, lineage B.1.1.7, and estimated its emergence back to September 2020 [4]. Among other notable genetic alterations that were found to increase viral binding affinity with angiotensin-converting-enzyme 2 receptor (N501Y) [5] or facilitate epithelial cell entry (P681H) [6], deleterious mutations at positions H69/V70 were the initial clue to the new lineage [7]. These deletions affected a widely used polymerase chain reaction assay, the TaqPathTM COVID-19 CE-IVD RT-PCR Kit (ThermoFisher, Luzern, Switzerland), preventing the amplification of the S-gene target, resulting in S-gene target failure (SGTF). While the TaqPath Kit targets three different SARS-CoV-2 genes, the Open Reading Frame 1ab (ORF1ab), the nucleocapsid (N), and the spike (S) gene, a positive result is given with the faultless amplification of two out of three target sequences. After the discovery of the SGTF signature, positive samples presenting without spike target signals then quickly became synonymous with the Alpha variant and were subsequently used as a proxy for its presence as the worldwide prevalence increased [7,8,9,10,11,12,13].

With the emergence of the B.1.617.2 lineage, designated as Delta by the WHO [14] and lacking the ΔH69/V70, triple-gene positives started to increase again in frequency after a long period of Alpha dominance, where more than 90% of samples presented with the SGTF signature [15,16]. While there were still a few SGTF-negative lineages in circulation at that time, an increase in triple-gene positives could nevertheless be attributed to the Delta variant, and the absence of the SGTF was subsequently used as a proxy for its proportion among the different lineages [15,16].

The emergence of the Omicron variants BA.1, BA.2, and BA.4/BA.5 then followed this alternating pattern of S-gene amplification presence and absence.

While the SGTF signature was extensively used as a surrogate for different VOCs during the pandemic, systematic studies on its accuracy and effectiveness as a surveillance tool are scarce. In this study, we therefore investigated the diagnostic accuracy of the absence and presence of the SGTF signature as a proxy for dominant variants throughout the pandemic in six regions of Switzerland and the principality of Liechtenstein.

## 2. Materials and Methods

### 2.1. Setting

Throughout the pandemic, Dr. Risch laboratories served all six regions of Switzerland as well as the principality of Liechtenstein with SARS-CoV-2 PCR testing. Referred samples originated from every canton and were mostly nasopharyngeal swabs or saliva samples. Alongside other testing methods, routine PCR testing was performed using the TaqPath COVID-19 CE-IVD RT-PCR Kit by ThermoFisher Scientific, Lucerne, Switzerland (TaqPath). All positive samples, starting from calendar week 37 of 2020 up to calendar week 47 of 2022, tested with the TaqPath Kit were included in the study, spanning a period encompassing the SARS-CoV-2 variant waves of B.1.1.7 (Alpha), B.1.617.2 (Delta), as well as Omicron variants BA.1, BA.2, and BA.4/5. Omicron variants BA.4 and BA.5 were summarized due to their concurrent presence and identical SGTF pattern.

### 2.2. SARS-CoV-2 RT-PCR

The TaqPath Kit was used in a high-throughput manner, and tests were performed according to the manufacturer’s instructions with either the QuantstudioTM 5 or the QuantstudioTM 7 qPCR System (Amplitude Solution) using the MagMax Viral/Pathogen Nucleic Acid Isolation Kit (ThermoFisher Scientific, Lucerne, Switzerland). Only samples tested using the TaqPath Kit were included in this study. This kit targets the genes for ORF1ab, the nucleocapsid protein (N-gene), as well as the spike protein (S-gene). As per the manufacturer’s instructions, samples were reported as positive when the detection of at least two out of these three target genes was achieved with a cycle threshold (Ct) value less than 37.

### 2.3. S-Gene Target Failure (SGTF) Definition

As a diagnostic criterium, complete S-gene target failure (cSGTF) was defined as the absence of S-gene target amplification in the presence of the faultless detection of ORF1ab- and N-gene amplicons. A partial SGTF (pSGTF) was then specified to present a diminished amplification of the S-gene (higher Ct-value) compared to the coherent detection of ORF1ab- and N-gene amplicons. For this, the S-gene shift was calculated for each SGTF-negative sample as follows:

S-gene shift = CT (S) − CT (ORF1ab)(1)

Samples exhibiting a significant S-gene shift were specified as pSGTF, and an empirical approach was used to differentiate between unspecific pSGTF and “real” pSGTF to establish a reasonable cutoff value for sensitivity and specificity calculations.

### 2.4. Confirmation of Variants of Concern (VOCs)

Only samples with a Ct value < 30 were considered for analysis. This cutoff was defined as such because (i) samples with Ct > 30 have the potential to produce unspecific target failures in any of the three target amplifications (see Supplementary Material Figure S1), and (ii) whole-genome sequencing with adequate coverage is rarely achievable in samples with Ct > 30. Variant confirmation was then performed with mutation-specific PCR (VirSNiP, TIB MOLBIOL, Berlin, Germany) or whole-genome sequencing (WGS). WGS was performed using a GridIon nanopore sequencer (Oxford Nanopore Technologies, Oxford, UK) for approximately 24 h and according to the manufacturer’s instructions using the ARTIC and Midnight protocols [17]. Amplification was performed with the most recent primer versions to address potential sequence dropouts due to the evolution of the viral genome. Analysis was based on the ARTIC pipeline. All genomic sequences were analyzed using the Pangolin COVID-19 Lineage Assigner (version v4.3, pangolin-data version v1.20) [18] based on the Pangolin nomenclature [19]. The VirSNiP Assays used are listed in Table 1.

Since all dominant SARS-CoV-2 variants in this time span showed an alternating pattern of SGTF presence and absence, variant confirmation was performed when the frequency of either one started to increase again after the steady-state period of the previously dominant variant (e.g., with the Alpha variant being dominant during the beginning of 2021, stable frequencies of SGTF at a high level were observed. The emergence of the Delta variant, however, increased the number of samples without SGTF, which triggered variant confirmation by decreasing the frequency of SGTF). Frequency surveillance was performed on a weekly basis.

### 2.5. Logistic Growth Rates and Sigmoid’s Midpoint

For a comparison of the different variant waves, a logistic model was fitted to the data corresponding to the introduction of each variant. In this case, Sigmoid’s midpoint (t_0_) describes the time value (*x*-axis) where 50% of the circulating virus belongs to the variant of interest, while the logistic growth rate (α) describes the slope at this point. The parameters α and t_0_ were estimated according to Chen et al. [21] with help of the nonlinear regression function in MedCalc^®^ statistical software v20.027 according to the equation for logistic regression:

(2) $$y(t)=\frac{1}{1+e^{-a*\left(t-t_{0}\right)}}$$

where α is the logistic growth rate, and t_0_ is the sigmoid’s midpoint.

A logistic model was fitted to the frequency of SGTF per week for each SGTF-positive variant (i.e., Alpha, Omicron BA.1, and Omicron BA.4/BA.5). Inversely, 1-SGTF was used as a basis for a logistic model fitted to SGTF-negative variants (i.e., Delta and Omicron BA.2). With the help of the above logistic regression formula, α and t_0_ were estimated. The models were calculated over the periods reported in Table 2.

### 2.6. Data Collection and Analysis

According to Article 2 of the Swiss Federal Act on Research involving Human Beings, an analysis on anonymized biological material and anonymized health data does not qualify as research in a strict sense of the law, and approval of a cantonal ethics commission as well as informed consent can thus be waived. Only data pertaining to sampling date and time, as well as the sampling place (canton) corresponding to the sampled materials, were used for this analysis, meaning our study was conducted without the use of any personal information of patients.

Descriptive statistics were obtained using Microsoft Excel v2312(Microsoft, Seattle, WA, USA), while Medcalc (Mariakerke, Belgium) was used for computations.

### 2.7. Comparison with GISAID

As the main goal of this study was to evaluate the effectiveness of the SGTF as a tool for estimating the proportion of different SARS-CoV-2 variants in real time, we compared our data to data from the Global Initiative on Sharing All Influenza Data (GISAID), as accumulated sequencing results on GISAID are most likely the best approximation to real-world variant proportion.

For this, CoV-Spectrum, enabled by data from GISAID, was accessed on the 27 February 2023. We only searched for data from Switzerland. The results for B.1.1.529.4* and B.1.1.529.5* were combined, as both exhibit the SGTF and were collectively responsible for the corresponding wave. The search terms and corresponding time periods used are presented in Table 3.

Subsequently, data for proportion from the “sequences over time” and the “international comparison” tab were downloaded and used for comparison.

## 3. Results

### 3.1. General

During the study period, from week 37 of 2020 to week 47 of 2022, a total of 2,488,446 unique samples were tested via PCR for SARS-CoV-2, of which 313,698 were positive (positivity: 12.61%; 95% confidence interval [CI], 12.56% to 12.65%). In total, 272,058 (86.7%) of the positive samples were detected using the TaqPath Kit, and 240,896 (88.5%) had Ct values < 30 for the ORF1ab target gene and were therefore included in the study. The sample materials were mainly nasopharyngeal swabs (81.8%) and saliva in standardized NaCl solution (15.8%). Of these positives, 120,155 (49.9%) were SGTF-negative (i.e., detection of at least the ORF1ab- and S-gene targets, compatible with WT, Delta, and Omicron BA.2 variants), and 120,741 (50.1%) were SGTF-positive (i.e., detection of the ORF1ab- and N-genes, compatible with B.1.258, Alpha, BA.1, and BA.4/BA.5). No significant difference was observed between nasopharyngeal swabs and saliva samples, as 50.2% and 49.0%, respectively, presented with SGTF. Missing N-gene targets (with the detection of ORF1ab- and S-genes) were observed in 0.09% of samples. These were confined to the Delta variant, and causal mutations have been discussed elsewhere [22,23,24]. Missing ORF1ab-gene targets were not observed.

### 3.2. The SGTF Oscillator

**Figure 1:**
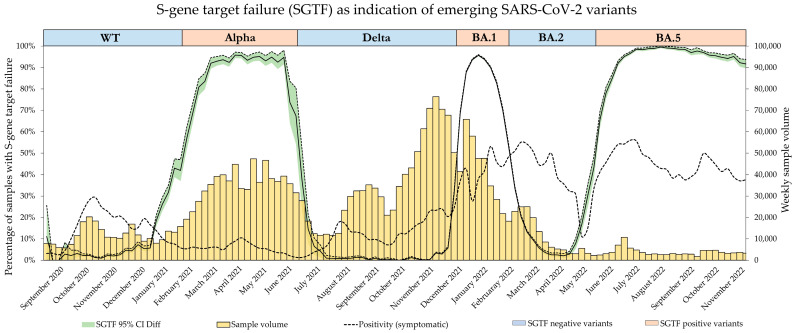
Frequency of samples presenting with complete S-gene target failure (cSGTF) in the TaqPath Kit over a period of 2 years. The SGTF was used to estimate the proportion of emerging SARS-CoV-2 variants. Black straight line: the frequency of samples with SGTF with 95% CI is shown via the green shaded area. Black dotted line: weekly positivity rate. Yellow bars: weekly sample volume.

The distinction between the SGTF-positive and SGTF-negative samples resulted in an oscillating indicator. As the SGTF indicates SARS-CoV-2 variants harboring ΔH69/V70, the SGTF frequency directly correlated with the proportion of these variants, while the inverse was true for variants without the deletion. During the study period, dominant SARS-CoV-2 variants alternated between wild type and deletion at this position, resulting in an oscillating frequency for SGTF, as depicted in Figure 1. Although the SGTF is not variant-specific, its frequency was usually tantamount to the dominant or emerging variant, as other variants had a very low prevalence. Sample volume was highest during the switch from Delta to BA.1, while positivity was highest during the Omicron BA.1 and BA.2 waves. A switch from an SGTF-negative variant to an SGTF-positive variant was accompanied by a decrease in Ct value from ~30 to 20 for samples with SGTF, indicating a change from unspecific SGTF in low-viral-load samples to specific SGTF in high-viral-load samples during the expansion of new SGTF-positive variants (Figure S2). 

### 3.3. S-Gene Shift

**Figure 2:**
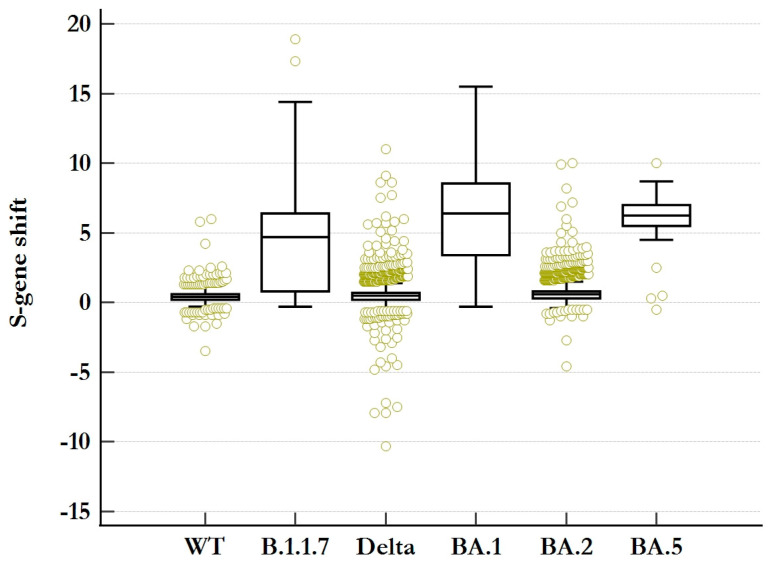
Boxplot analysis of the S-gene shift values of all triple-gene positive VOC-determined samples. The S-gene shift for each sample was calculated as follows: Ct (S-gene)—Ct (ORF1ab). While variants without ΔH69/V70 typically present as triple-gene positives with S-gene shift < 1, triple-gene positives of variants that acquired the deletion would typically show an S-gene shift > 1.

The three target genes in the TaqPath Kit typically generate similar Ct values in a positive sample and rarely show a difference > 1 Ct value. While variants that acquired ΔH69/V70 mostly led to complete SGTF, there were still 12,124 (10.04%) SGTF-negative samples presenting with an S-gene shift > 1 Ct value, a delayed amplification of the S-gene target, while 804 (0.67%) SGTF-negative samples presenting with an S-gene shift > 5 Ct values were identified. A difference of up to three Ct values is within the cycle threshold variability of different targets within a single test performance [25], but greater differences are, in most cases, results of SGTF-positive samples rather than problems with the assay. This is illustrated in Figure 2, which shows the distribution of the S-gene shift among all samples with the amplification of all three target genes for all dominant variants. The mean values for the S-gene shift of the B.1.1.7, BA.1, and BA.5 variants were 4.6, 6.1, and 5.9 Ct-values, respectively, while the means for the SGTF-negative variants were below one. WGS did not reveal any additional mutations other than ΔH69/V70, which might be responsible for an increase in S-gene shift (pSGTF). The occurrence of pSGTF was, however, found to increase with higher viral load/lower Ct-values (Figure S4).

### 3.4. Sensitivity and Specificity of S-Gene Target Failure

#### 3.4.1. General

VOC confirmation has been successfully performed on 17,724 samples, corresponding to 7.4% of all samples included in the study. A total of 10,032 samples (56.6%) were confirmed using SNPs, while WGS was performed on 7692 samples (43.4%). Overall sensitivity and specificity, including VOC determination by either whole-genome sequencing or mutation-specific PCR, was calculated to be 93.2% [92.7%, 93.7%] and 99.3% [99.2%, 99.5%], respectively, based on 17,724 VOC-confirmed samples (Table 4). The isolated sensitivity and specificity values for mutation-specific PCR were 90.2% [89.4%, 91.0%] and 99.8% [99.6%, 99.9%] (Table S6), respectively, and 98.3% [97.8%, 98.7%] and 98.9% [98.5%, 99.2%], respectively, for WGS confirmation (Table S7).

In total, 14′784 samples were confirmed by either WGS or mutation-specific PCR during the defined periods of variant emergence. For the Alpha/B.1.258 variant, 3628 SGTF-positive (3615 Alpha/13 non-Alpha) and 1724 SGTF-negative (66 Alpha/1658 non-Alpha) samples were confirmed, cumulating in a sensitivity of 98.2% [97.7%, 98.6%] and a specificity of 99.2% [98.7%, 99.6%] (Table S1). During the emergence of the Omicron BA.1 variant, 2999 SGTF-positive (2994 BA.1, 5 non-BA.1) and 1423 SGTF-negative (193 BA.1, 1230 non-BA.1) samples were confirmed with a sensitivity of 93.9% [93.1%, 94.8%] and a specificity of 99.6% [99.1%, 99.9%] (Table S3). For the Omicron BA.4/BA.5 wave, 478 SGTF-positive (457 BA.5, 21 non-BA.5) and 343 SGTF-negative (3 BA.5, 340 non-BA.5) samples were confirmed, resulting in a sensitivity of 99.4% [98.1%, 99.9%] and a specificity of 94.2% [91.3%, 96.4%] (Table S5). 

For the SGTF-negative Delta and Omicron BA.2 variants, 10 SGTF-positive (non-Delta) and 680 SGTF-negative (679 Delta, 1 non-Delta) samples and 846 SGTF-positive (2 BA.2, 844 non-BA.2) and 2653 SGTF-negative (2230 BA.2, 423 non-BA.2) samples were confirmed, respectively (Tables S2 and S4). Sensitivity and specificity were 100.00% [99.46% to 100.00%] and 90.91% [58.72% to 99.77%] for the Delta variant and 99.91% [99.68% to 99.99%] and 66.61% [63.94% to 69.21%] for Omicron BA.2, respectively.

#### 3.4.2. Implementing a Cutoff for Partial SGTF

**Figure 3:**
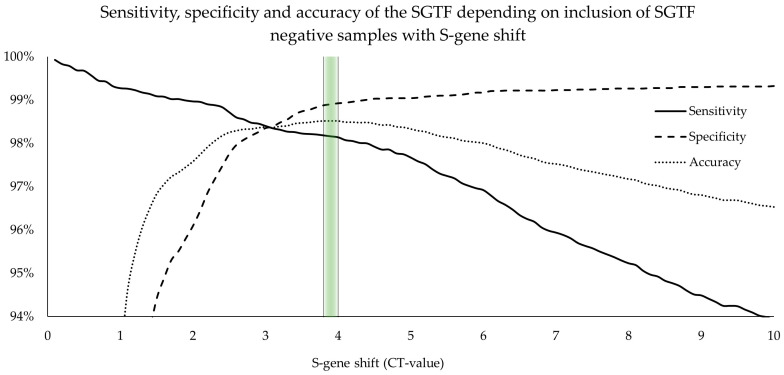
Empirical approach to improve the sensitivity and specificity of the SGTF depending on the extent of the S-gene shift in SGTF-negative samples. Sensitivity and specificity were calculated as 93.0% [92.4%, 93.5%] and 95.8% [95.3%, 96.2%], respectively, with the sole inclusion of SGTF-positive samples. Green color bar: Highest value for accuracy.

Samples with elevated S-gene shift (pSGTF) usually belong to SGTF-positive variants and should therefore be considered as such. Consequently, we tried to improve the previously determined accuracy for complete SGTF of 96.2% [95.9%, 96.5%] by determining the best cutoff for S-gene shift using an empirical approach. As seen in Figure 3, a S-gene shift of 3.8–4 Ct-values produces the best value for accuracy (98.5%, 98.3% to 98.7%) with a sensitivity and specificity of 98.2% [97.9% to 98.4%] and 98.9% [98.6% to 99.1%], respectively (Table 4). Considering SGTF-negative samples with S-gene shift > 3.8 (pSGTF) as SGTF-positive therefore increases overall sensitivity by 5.0% while reducing specificity by 0.4% (Table 4).

As for the overall sensitivity and specificity, variant-specific performance characteristics were improved when samples with partial SGTF were included (Table 5). Sensitivity and specificity were improved to 99.3% [98.9%, 99.5%] and 99.0% [98.4%, 99.4%] for the Alpha variant, 99.5% [99.2%, 99.7%] and 99.3% [98.6%, 99.7%] for Omicron BA.1, and 100% [99.2%, 100%] and 94.2% [91.3%, 96.4%] for the Omicron BA.4/BA.5 wave.

### 3.5. Estimating Logistic Growth Rates (α) and Sigmoid’s Midpoint (t_0_) for Each Variant Wave

While the sensitivity and specificity of the SGTF assess the method-specific correct identification of ΔH69/V70 in a viral sequence, they do not give an assessment of the predictive value of variant proportion and do not assess variant dynamics. Because multiple “smaller” variants with SGTF have existed simultaneously to the respective dominant variants, agreement among the percentage of SGTF and proportion of the dominant variant is highly dependent on the prevalence of these “smaller” variants.

To assess the accuracy of the SGTF signature in predicting variant proportion and dynamics, estimates for α and t_0_ based on SGTF data and WGS data from GISAID were compared. α and t_0_ were calculated for the whole dataset. Our calculations were based on SGTF data as a proxy and logistic regression analysis. 

**Figure 4:**
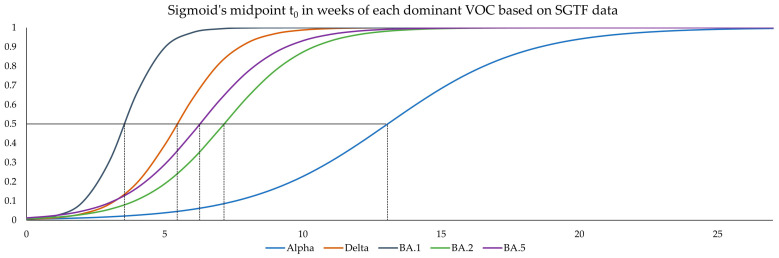
Sigmoid functions of all variant waves obtained by logistic regression. All functions are initialized at the same point to show relative differences. Black dotted lines: t_0_ of the respective variants.

Summarized α and t_0_ were calculated to be 0.40 [0.36, 0.44] and 13.06 [12.78, 13.35] for Alpha, −0.97 [1.14 to −0.81] and 5.45 [5.25, 5.65] for Delta, 1.49 [1.17, 1.80] and 3.54 [3.38, 3.70] for Omicron BA.1, −0.68 [ 0.74 to 0.62] and 7.14 [7.00, 7.29] for Omicron BA.2, and 0.70 [0.65, 0.75] and 6.27 [6.16, 6.38] for Omicron BA.5. Negative values for growth rates indicate VOCs without the spike mutation ΔH69/V70, for which the inverse, i.e., an increase in the fraction of SGTF-negative samples, was used as a proxy. The respective sigmoid curves, initialized at the same point in time to show relative differences, are shown in Figure 4. While t_0_ is highly dependent on the selected starting week of the underlying dataset, α was not significantly impacted by this, as can be viewed in Supplementary Material Figure S3, where we investigated the robustness of α and t_0_ depending on the initialization week for the Omicron BA.1 dataset.

### 3.6. Agreement between SGTF and Whole-Genome Sequencing Data

In comparison to data from GISAID, no significant difference was found for α or t_0_ for estimates based on SGTF data for the Alpha, Delta, BA.2, and BA.4/BA.5 variant waves, as 95% confidence intervals overlap in every case. The growth rates for BA.1 show a clear deviation, although 95% CIs still overlap (SGTF: 1.49 [1.17 to 1.80]; GISAID: 1.02 [0.79 to 1.25]), while Sigmoid’s midpoints were significantly different (SGTF: 3.54 [3.38 to 3.70]; GISAID: 3.06 [2.82 to 3.30]). Summarized data can be found in Table 6 and Table 7.

**Figure 5:**
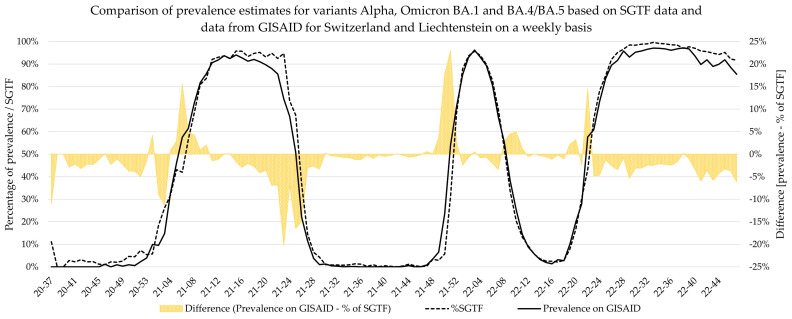
A comparison of proportion estimates based on SGTF data and data from GISAID for the SGTF-positive variants: Alpha, Omicron BA.1, and Omicron BA.4/BA.5.

**Figure 6:**
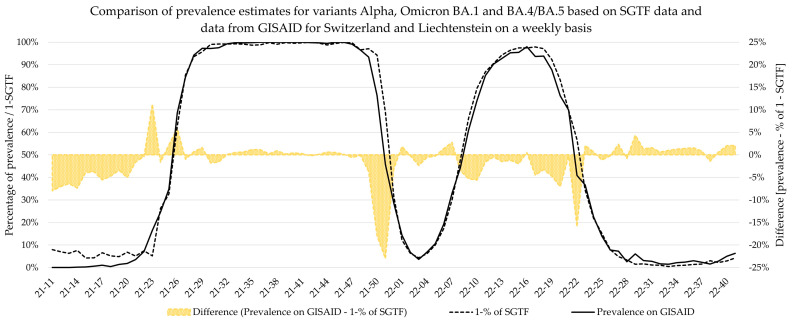
A comparison of proportion estimates based on SGTF data and data from GISAID for the SGTF-negative variants: Delta and Omicron BA.2.

For this comparison, parameters calculated based on GISAID only included variant-specific data. However, multiple variants that all exhibited the SGTF phenomenon (or not, for that matter) were in circulation simultaneously, and all of them have an impact on the percentage of SGTF at any given time. The differentiation of these variants based on the SGTF pattern is not possible. Therefore, these variants confound estimations for parameters such as growth rate and Sigmoid’s midpoint when based on SGTF data. In Figure 5 and Figure 6, this is most prominently visible during the emergence of the Delta variant, where the lines of the GISAID and the SGTF dataset clearly diverge during calendar weeks 14 and 26 in 2021.

## 4. Discussion

We extensively investigated the accuracy and effectiveness of the SGTF signature and its absence as a surrogate marker for the emergence of the Alpha and Delta variants, as well as the Omicron variants BA.1, BA.2, and BA.4/BA.5. We determined the sensitivity and specificity of complete SGTF to be 93.2% [92.7%, 93.7%] and 99.3% [99.2%, 99.5%], respectively. We have also shown that classifying SGTF-negative samples with S-gene shift (pSGTF) as complete SGTF increased sensitivity and defined the best cutoff value at pSGTF > 3.8 Ct-values. This has already been reported by Borges V et al. [26], but no clear cutoff value had been established previously. By establishing this cutoff value, accuracy, sensitivity, and specificity were improved to 98.5% [98.3%, 98.7%], 98.2% [97.9% to 98.4%], and 98.9% [98.6% to 99.1%], respectively.

Many publications have evaluated different approaches to determining the accuracy and applicability of the SGTF in variant detection [27,28,29,30,31,32,33]. In our study, the sensitivity and specificity values based on WGS VOC determination were in line with previously reported values [34]; our calculations based on variant-specific PCR, however, were consistently lower than for WGS, indicating a reduced accuracy in lineage classification. 

Additionally, weekly logistic growth rates and Sigmoid’s midpoint were estimated for each variant wave based on SGTF data and did not significantly differ to estimates based on comprehensive data from GISAID for the Alpha, Delta, BA.2, and BA.4/BA.5 variants. However, the estimates for Omicron variant BA.1 showed clear deviations for growth rate, while the estimates for Sigmoid’s midpoint were significantly different. This deviation is visible in Figure 5, as the BA.1 estimate based on SGTF data lags the GISAID estimate by 1 week during calendar weeks 49–51 of 2021. Although the samples analyzed in this study originated from all over Switzerland, the distribution was not equal among all regions and cantons, and it is possible that the very fast emergence of the Omicron BA.1 variant, coupled with potentially low testing coverage in certain regions, might have introduced bias in our data during this time. Generally, the variant proportion estimates based on SGTF or GISAID agreed well, and considering two weeks delay for sequencing results, the upside of using such a surrogate marker during a fast-paced pandemic seems obvious. This, however, is owed to the specific dynamics we witnessed during the SARS-CoV-2 pandemic, as dominant variants emerged quickly, while non-dominant variants had a very low prevalence in general. The highest proportion of non-dominant variants was observed during the Alpha period, where proportion estimates for SGTF and GISAID diverge the most. However, this did not have a significant impact on the sigmoid function and, therefore, the variant dynamics estimates. 

The greatest sample volume was experienced during the dominance of the Omicron BA.1 variant. Due to its short period of dominance, the Delta, Omicron BA.1, and Omicron BA.2 variants were present simultaneously at its peak. Since the SGTF-positive samples almost certainly belonged to the Omicron BA.1 variant, we extensively confirmed the SGTF-negative samples to distinguish between the vanishing Delta variant and the newly emerging Omicron BA.2 variant. The unprecedented number of samples has prompted us to favor mutation-specific PCR for VOC confirmation as it is less time-consuming than WGS. With the increase in samples, however, there was also an increase in samples presenting with pSGTF, and through the extensive VOC confirmation of the SGTF-negative samples, we introduced bias into our data. This is clearly noticeable in Table S4, where sensitivity and specificity for Omicron BA.2 were 100.0% [99.8%, 100.0%] and 5.5% [3.6%, 8.1%], respectively, for SNP confirmation, while WGS confirmation was in line with that of the other variants. This low specificity can therefore be explained by the extensive variant confirmation of the SGTF-negative samples during the period with the highest sample volume, leading to a relative increase in confirmed samples with S-gene shift. The increase in specificity to 70.9% [66.4%, 75.1%] for pSGTF > 3.8 supports this observation and shows the importance of distinguishing between true triple positives and pSGTF. Importantly, WGS did not reveal any additional mutations that might be causative for pSGTF. However, pSGTF was found to occur more frequently in high-viral-load samples, suggesting low frequent primer/probe mismatch if high numbers of S-gene amplicons are in the reaction. This observation is in agreement with previous reports from Portugal [26]. Further research is needed to precisely determine the molecular mechanism behind pSGTF.

The reasons behind the alternating pattern of SGTF and non-SGTF dominant variants remain inadequately understood, and no real evidence for as to why this switching was favored by viral evolution has been presented. In 2021, ΔH69/V70 in the spike glycoprotein was found to have arisen independently at least 13 times [35]. In vitro studies have demonstrated that this deletion does increase infectivity by higher levels of spike incorporation into virions and that it mediates faster fusion than the wild type [36]. It does not, however, significantly reduce the sensitivity of neutralizing antibodies from recovered individuals and is probably not an immune escape mechanism [37]. Recently, it was also reported that the SGTF can help identify the development of sub-lineages within a patient with persistent SARS-CoV-2 infection [38].

Our study has some limitations. First, we only investigated samples with relatively high viral load (Ct < 30); therefore, our study may be biased towards these types of samples. Second, while the TaqPath Kit was the main method used for SARS-CoV-2 PCR testing, testing was also performed with multiple other platforms; therefore, not all potential SGTF samples were included in the study. Third, the TaqPath Kit is a commercially available PCR assay that does not grant flexibility for primer design or primer binding regions or knowledge thereof. Therefore, only assumptions about the primer/probe binding sites as well as the location of ΔH69/V70 on these sites are possible. Based on our data, however, it seems that, at least for the S-gene, the test design allows for a certain amount of mismatch so that detectable PCR signals can still be produced with high numbers of S-gene amplicons in the reaction.

Importantly, this study also demonstrates that multiple target assays are critical in PCR diagnostics. Diagnostic targets may be under an evolutionary pressure if a lot of cases occur, and pattern observation can therefore help in observing diagnostic driven evolution.

In conclusion, in this paper, we have reported the SGTF signature as a surrogate marker with high predictability for SARS-CoV-2 variants that acquired ΔH69/V70. Since SGTF surveillance relies on a diagnostic RT-PCR test, it allows for faster turnaround times with higher throughput while being less expensive than genomic sequencing. It is limited by the fact that no mutations other than ΔH69/V70 are being assessed, and specific lineage classification by WGS is therefore still required. It also relies on a continued alternating pattern of SGTF-positive and SGTF-negative dominant variants. Despite its limitations, the SGTF signature allowed us to make exceptionally accurate and fast predictions about the changing dynamics of the SARS-CoV-2 pandemic. Continued monitoring in combination with genomic sequencing will likely prove useful in the future. 

## Figures and Tables

**Table 1:** VirSNip SARS-CoV-2 assays (TIB MOLBIOL) targeting variant-specific single-nucleotide polymorphisms that were used to differentiate between concurrent variants [20].

VirSNiP	SARS-CoV-2 Spike Assays
N501Y	Differentiation B.1.1.7/B.1.258
E484K	Differentiation wild type/B.1.351 + P.1
V1176F	Differentiation B.1.351/P.1
L452R	Screening for B.1.617.2
S478K	Screening for B.1.617.2
S371L S373P L452R	Differentiation B.1.617.2/BA.1/BA.2

**Table 2:** Initialization and endpoints used for the estimation of growth rates (α) and Sigmoid’s midpoint (t_0_) for each SARS-CoV-2 variant.

Variant	Initialization	Endpoint
B.1.1.7 (Alpha)	16 November 2020	23 May 2021
B.1.617.2 (Delta)	24 May 2021	22 August 2021
B.1.1.529.1 (BA.1)	6 December 2021	23 January 2022
B.1.1.529.2 (BA.2)	10 January 2022	10 April 2022
B.1.1.529.4 (BA.4) + B.1.1.529.5 (BA.5)	25 April 2022	24 July 2022

**Table 3:** Search terms and corresponding time periods used to download GISAID-enabled proportion data for all dominant SARS-CoV-2 variants.

Search Term	From	To
B.1.1.7*	16 November 2020	23 May 2021
B.1.617.2*	24 May 2021	22 August 2021
B.1.1.529.1*	6 December 2021	23 January 2022
B.1.1.529.2*	10 January 2022	10 April 2022
B.1.1.529.4*	25 April 2022	24 July 2022
B.1.1.529.5*	25 April 2022	24 July 2022

* Includes all related sub-lineages

**Table 4:** Sensitivity and specificity values of the S-gene target failure (SGTF) signature without and with the inclusion of SGTF-negative samples with S-gene shift > 3.8 CT values.

	VOC Determination (WGS and Mutation-Specific PCR)					
	TaqPath	SGTF VOC *	nSGTF VOC **	Total		
cSGTF	SGTF-positive	8561	56	8617	Sensitivity [95% CI]	93.2% [92.7%, 93.7%]
	SGTF-negative	621	8486	9107	Specificity [95% CI]	99.3% [99.2%, 99.5%]
	Total	9182	8542		Accuracy [95% CI]	96.2% [95.9%, 96.5%]
pSGTF > 3.8	SGTF-positive	9013	94	9107	Sensitivity [95% CI]	98.2% [97.9%, 98.4%]
	SGTF-negative	169	8448	8617	Specificity [95% CI]	98.9% [98.6%, 99.1%]
	Total	9182	8542		Accuracy [95% CI]	98.5% [98.3%, 98.7%]

* Variants—B.1.258, Alpha, BA.1, BA.4/BA.5. ** Variants—WT, Delta, BA.2

**Table 5:** Variant-specific sensitivity and specificity including variant confirmation with whole-genome sequencing (WGS) or mutation-specific PCR (SNP). The parameters were improved by the inclusion of pSGTF samples with S-gene shift > 3.8.

	cSGTF		pSGTF > 3.8	
Variants	Sensitivity [95% CI]	Specificity [95% CI]	Sensitivity [95% CI]	Specificity [95% CI]
B.1.1.7* (Alpha)	98.2 [97.7, 98.6]	99.2 [98.7, 99.6]	99.2 [98.9, 99.5]	99.0 [98.4, 99.4]
B.1.617.2* (Delta)	100.0 [99.5, 100.0]	90.9 [58.7, 99.8]	99.7 [98.9, 100.0]	100.0 [71.5, 100.0]
B.1.1.529.1* (BA.1)	93.9 [93.1, 94.8]	95.9 [94.6, 96.9]	99.5 [99.2, 99.7]	99.3 [98.6, 99.7]
B.1.1.529.2* (BA.2)	99.9 [99.7, 100.0]	66.6 [63.9, 69.2]	99.3 [98.8, 99.6]	89.5 [87.7, 91.1]
B.1.1.529.4* (BA.4)/B.1.1.529.5* (BA.5)	99.4 [98.1, 99.9]	94.2 [91.3, 96.4]	100.0 [99.2, 100.0]	94.2 [91.3, 96.4]

* Includes all related sub-lineages

**Table 6:** Growth rates and Sigmoid’s midpoints calculated based on data from GISAID for Switzerland.

GISAID	Alpha	Delta	BA.1	BA.2	BA.4/BA.5
Growth rate	0.46	0.91	1.02	0.63	0.62
Std Error	0.032	0.066	0.09	0.02	0.05
95% CI	0.39 to 0.52	0.76 to 1.05	0.79 to 1.25	0.59 to 0.66	0.51 to 0.72
Sigmoid’s midpoint	12.83	5.30	3.06	7.32	6.22
Std Error	0.17	0.09	0.09	0.05	0.14
95% CI	12.47 to 13.18	5.10 to 5.50	2.82 to 3.30	7.22 to 7.42	5.90 to 6.53

**Table 7:** Growth rates and Sigmoid’s midpoint calculated from SGTF data.

SGTF Proxy	Alpha	Delta	BA.1	BA.2	BA.4/BA.5
Growth rate	0.40	0.97	1.49	0.68	0.70
Std Error	0.02	0.08	0.12	0.03	0.02
95% CI	0.36 to 0.44	0.81 to 1.14	1.17 to 1.80	0.62 to 0.74	0.65 to 0.75
Sigmoid’s midpoint	13.06	5.45	3.54	7.14	6.27
Std Error	0.14	0.09	0.06	0.07	0.05
95% CI	12.78 to 13.35	5.25 to 5.65	3.38 to 3.70	7.00 to 7.29	6.16 to 6.38

## Data Availability

Data are contained within the article and Supplementary Materials.

## Supplementary Materials

The following supporting information can be downloaded at: https://www.mdpi.com/article/10.3390/microorganisms12020321/s1, Figure S1. The ratio of samples presenting with S-gene target failure (SGTF) or N-gene target failure (NGTF) in relation to triple-positive samples. An increasing number of samples exhibiting SGTF or NGTF is observed when cycle threshold (CT) values for the ORF1ab target gene are greater than 30, as illustrated by the rising slope; Figure S2. Weekly mean ORF1AB Ct-value of SGTF positive samples. The frequency of SGTF is plotted to compare to the emergence of new dominant variants. Depicted is the change from unspecific SGTF with Ct-value ~30 in low viral load samples to specific SGTF with Ct value ~20 in high viral load samples during the expansion of new SGTF positive variants; Figure S3. Sensitivity analysis for growth rate and sigmoid’s midpoint depending on initialization of logistic fit to the dataset. The growth rate does not change significantly when the initialization point is moved in one-week intervals (a), while Sigmoid’s midpoint changes accordingly (b). Depicted is the logistic fit for the Omicron BA.1 dataset, as this dataset is the least robust with the least datapoints due to its short emergence period; Table S1. B.1.1.7* (alpha) specific sensitivity and specificity; Table S2. B.1.617.2* (delta)) specific sensitivity and specificity; Table S3. B.1.1.529.1* (BA.1) specific sensitivity and specificity; Table S4. B.1.1.529.2* (BA.2) specific sensitivity and specificity; Table S5. B.1.1.529.4*/B.1.1.529.5* (BA.4/BA.5) specific sensitivity and specificity; Table S6. Sensitivity and specificity based on VOC determination with mutation specific PCR (SNP); Table S7. Sensitivity and specificity based on VOC determination with whole-genome-sequencing (WGS); Table S8. Dominant SARS-CoV-2 variants and the respective amount of samples identified in this study by either whole-genome sequencing (WGS) or mutation specific PCR (SNP).

## References

[1] WHO Coronavirus (COVID-19) Dashboard|WHO Coronavirus (COVID-19) Dashboard with Vaccination Data. https://covid19.who.int/.

[2] World Health Organization. https://www.who.int/docs/default-source/coronaviruse/situation-reports/20200121-sitrep-1-2019-ncov.pdf.

[3] CDC Coronavirus Disease 2019 (COVID-19). https://www.cdc.gov/coronavirus/2019-ncov/variants/variant-classifications.html.

[4] Public Health England (2020). Investigation of Novel SARS-CoV-2 Variant—Variant of Concern 202012/01.

[5] Starr T.N., Greaney A.J., Hilton S.K., Ellis D., Crawford K.H.D., Dingens A.S., Navarro M.J., Bowen J.E., Tortorici M.A., Walls A.C. (2020). Deep Mutational Scanning of SARS-CoV-2 Receptor Binding Domain Reveals Constraints on Folding and ACE2 Binding. Cell.

[6] Hoffmann M., Kleine-Weber H., Pöhlmann S. (2020). A Multibasic Cleavage Site in the Spike Protein of SARS-CoV-2 Is Essential for Infection of Human Lung Cells. Mol. Cell.

[7] Walker A.S., Vihta K.-D., Gethings O., Pritchard E., Jones J., House T., Bell I., Bell J.I., Newton J.N., Farrar J. (2021). Tracking the Emergence of SARS-CoV-2 Alpha Variant in the United Kingdom. N. Engl. J. Med..

[8] Scobie H.M., Ali A.R., Shirk P., Smith Z.R., Paul P., Paden C.R., Hassell N., Zheng X.-Y., Lambrou A.S., Kondor R. (2023). Spike Gene Target Amplification in a Diagnostic Assay as a Marker for Public Health Monitoring of Emerging SARS-CoV-2 Variants—United States, November 2021–January 2023. MMWR Morb. Mortal. Wkly. Rep..

[9] Volz E., Mishra S., Chand M., Barrett J.C., Johnson R., Geidelberg L., Hinsley W.R., Laydon D.J., Dabrera G., O’Toole Á. (2021). Assessing transmissibility of SARS-CoV-2 lineage B.1.1.7 in England. Nature.

[10] Washington N.L., Gangavarapu K., Zeller M., Bolze A., Cirulli E.T., Schiabor Barrett K.M., Larsen B.B., Anderson C., White S., Cassens T. (2021). Emergence and rapid transmission of SARS-CoV-2 B.1.1.7 in the United States. Cell.

[11] Washington N.L., Gangavarapu K., Zeller M., Bolze A., Cirulli E.T., Barrett K.M.S., Larsen B.B., Anderson C., White S., Cassens T. (2021). Genomic epidemiology identifies emergence and rapid transmission of SARS-CoV-2 B.1.1.7 in the United States. medRxiv.

[12] Vogels C.B.F., Breban M.I., Ott I.M., Alpert T., Petrone M.E., Watkins A.E., Kalinich C.C., Earnest R., Roth-man J.E., Goes de Jesus J. (2021). Multiplex qPCR discriminates variants of concern to enhance global surveillance of SARS-CoV-2. PLoS Biol..

[13] Goncalves Cabecinhas A.R., Roloff T., Stange M., Bertelli C., Huber M., Ramette A., Chen C., Nadeau S., Gerth Y., Yerly S. (2021). SARS-CoV-2 N501Y Introductions and Transmissions in Switzerland from Beginning of October 2020 to February 2021—Implementation of Swiss-Wide Diagnostic Screening and Whole Genome Sequencing. Microorganisms.

[14] Tracking SARS-CoV-2 Variants. https://www.who.int/activities/tracking-SARS-CoV-2-variants.

[15] Hôpitaux Universitaires Genève. https://www.hug.ch/sites/interhug/files/structures/laboratoire_de_virologie/documents/Rapport%20hebdomadaire%20variants%20National/national-surveillance-variants-aug_-final.pdf.

[16] Chen C., Nadeau S., Yared M., Voinov P., Xie N., Roemer C., Stadler T. (2022). CoV-Spectrum: Analysis of globally shared SARS-CoV-2 data to identify and characterize new variants. Bioinformatics.

[17] Freed N.E., Vlková M., Faisal M.B., Silander O.K. (2020). Rapid and inexpensive whole-genome sequencing of SARS-CoV-2 using 1200 bp tiled amplicons and Oxford Nanopore Rapid Barcoding. Biol. Methods Protoc..

[18] O’Toole Á., Scher E., Underwood A., Jackson B., Hill V., McCrone J.T., Colquhoun R., Ruis C., Abu-Dahab K., Taylor B. (2021). Assignment of epidemiological lineages in an emerging pandemic using the pangolin tool. Virus Evol..

[19] Rambaut A., Holmes E.C., O’Toole Á., Hill V., McCrone J.T., Ruis C., Du Plessis L., Pybus O.G. (2020). A dynamic nomenclature proposal for SARS-CoV-2 lineages to assist genomic epidemiology. Nat. Microbiol..

[20] COVID-19—TIB MOLBIOL. https://www.tib-molbiol.de/de/covid-19.

[21] Chen C., Nadeau S.A., Topolsky I., Manceau M., Huisman J.S., Jablonski K.P., Fuhrmann L., Dreifuss D., Jahn K., Beckmann C. (2021). Quantification of the spread of SARS-CoV-2 variant B.1.1.7 in Switzerland. Epidemics.

[22] Ziegler K., Steininger P., Ziegler R., Steinmann J., Korn K., Ensser A. (2020). SARS-CoV-2 samples may escape detection because of a single point mutation in the N gene. Eurosurveillance.

[23] Alkhatib M., Bellocchi M.C., Marchegiani G., Grelli S., Micheli V., Stella D., Zerillo B., Carioti L., Svicher V., Rogliani P. (2022). First Case of a COVID-19 Patient Infected by Delta AY.4 with a Rare Deletion Leading to a N Gene Target Failure by a Specific Real Time PCR Assay: Novel Omicron VOC Might Be Doing Similar Scenario?. Microorganisms.

[24] Hilti D., Wehrli F., Roditscheff A., Risch M., Risch L., Egli A., Bodmer T., Wohlwend N. (2023). SARS-CoV-2 Nucleocapsid Protein Mutations Found in Switzerland Disrupt N-Gene Amplification in Commonly Used Multiplex RT-PCR Assay. Pathogens.

[25] Rhoads D., Peaper D.R., She R.C., Nolte F.S., Wojewoda C.M., Anderson N.W., Pritt B.S. (2021). College of American Pathologists (CAP) Microbiology Committee Perspective: Caution Must Be Used in Interpreting the Cycle Thresh-old (Ct) Value. Clin. Infect. Dis..

[26] Borges V., Sousa C., Menezes L., Gonçalves A.M., Picão M., Almeida J.P., Vieita M., Santos R., Silva A.R., Costa M. (2021). Tracking SARS-CoV-2 lineage B.1.1.7 dissemination: Insights from nationwide spike gene target failure (SGTF) and spike gene late detection (SGTL) data, Portugal, week 49 2020 to week 3 2021. Eurosurveillance.

[27] Bal A., Destras G., Gaymard A., Stefic K., Marlet J., Eymieux S., Regue H., Semanas Q., d’Aubarede C., Billaud G. (2021). Two-step strategy for the identification of SARS-CoV-2 variant of concern 202012/01 and other variants with spike deletion H69-V70, France, August to December 2020. Eurosurveillance.

[28] Carattini Y.L., Griswold A., Williams S., Valiathan R., Zhou Y., Shukla B., Abbo L.M., Parra K., Jorda M., Nimer S.D. (2023). Combined Use of RT-qPCR and NGS for Identification and Surveillance of SARS-CoV-2 Variants of Concern in Residual Clinical Laboratory Samples in Miami-Dade County, Florida. Viruses.

[29] Fontana-Maurell M., Motta F.D.C., Arruda M.B., Cardoso P., Ribeiro M., Andrade E., Godoy D.T., Costa E., Rocha D., Siqueira M.A.M. (2023). A straightforward one-step strategy for SARS-CoV-2 diagnosis and screening of variants of concern: A multicentre study. Mem. Inst. Oswaldo Cruz.

[30] Guerrero-Preston R., Rivera-Amill V., Caraballo K., Rodríguez-Torres S., Purcell-Wiltz A., García A.A., Torres R.S., Zamuner F.T., Zanettini C., MacKay M.J. (2022). Precision health diagnostic and surveillance network uses S gene target failure (SGTF) combined with sequencing technologies to track emerging SARS-CoV-2 variants. Immun. Inflamm. Dis..

[31] Mertens J., Coppens J., Loens K., Le Mercier M., Xavier B.B., Lammens C., Vandamme S., Jansens H., Goossens H., Matheeussen V. (2022). Monitoring the SARS-CoV-2 pandemic: Screening algorithm with single nucleotide polymorphism detection for the rapid identification of established and emerging variants. Clin. Microbiol. Infect..

[32] Migueres M., Lhomme S., Trémeaux P., Dimeglio C., Ranger N., Latour J., Dubois M., Nicot F., Miedouge M., Mansuy J.M. (2021). Evaluation of two RT-PCR screening assays for identifying SARS-CoV-2 variants. J. Clin. Virol..

[33] Subramoney K., Mtileni N., Bharuthram A., Davis A., Kalenga B., Rikhotso M., Maphahlele M., Giandhari J., Naidoo Y., Pillay S. (2022). Identification of SARS-CoV-2 Omicron variant using spike gene target failure and geno-typing assays, Gauteng, South Africa, 2021. J. Med. Virol..

[34] McMillen T., Jani K., Robilotti E.V., Kamboj M., Babady N.E. (2022). The spike gene target failure (SGTF) genomic signature is highly accurate for the identification of Alpha and Omicron SARS-CoV-2 variants. Sci. Rep..

[35] McCarthy K.R., Rennick L.J., Nambulli S., Robinson-McCarthy L.R., Bain W.G., Haidar G., Duprex W.P. (2021). Re-current deletions in the SARS-CoV-2 spike glycoprotein drive antibody escape. Science.

[36] Meng B., Kemp S.A., Papa G., Datir R., Ferreira I.A.T.M., Marelli S., Harvey W.T., Lytras S., Mohamed A., Gallo G. (2021). Recurrent emergence of SARS-CoV-2 spike deletion H69/V70 and its role in the Alpha variant B.1.1.7. Cell Rep..

[37] Lythgoe K.A., Golubchik T., Hall M., House T., Cahuantzi R., MacIntyre-Cockett G., Fryer H., Thomson L., Nurtay A., Ghafani M. (2023). Lineage replacement and evolution captured by 3 years of the United Kingdom Coronavirus (COVID-19) Infection Survey. Proc. Biol. Sci..

[38] Gandhi R.T., Castle A.C., de Oliveira T., Lessells R.J. (2023). Case 40-2023: A 70-Year-Old Woman with Cough and Shortness of Breath. N. Engl. J. Med..

