# Supplementary material for: S-Gene Target Failure as an Effective Tool for Tracking the Emergence of Dominant SARS-CoV-2 Variants in Switzerland and Liechtenstein, Including Alpha, Delta, and Omicron BA.1, BA.2, and BA.4/BA.5
Source: Microorganisms. 2024 Feb 3;12(2):321. doi: 10.3390/microorganisms12020321 (PMC10892681; doi:10.3390/microorganisms12020321)
Supplement: Supplementary file 1 [file microorganisms-12-00321-s001.zip › microorganisms-2828707-supplementary.pdf]

Supplementary material

Additional information regarding the calculation of sensitivity, specificity and accuracy

Sensitivity, specificity and accuracy were calculated using the MedCalc statistical software where the parameters are defined as:

$$Sensitivity = \frac{True\ Positives}{True\ Positives + False\ Negatives}$$

$$Specificity = \frac{True\ Negatives}{True\ Negatives + False\ Positives}$$

$$Accuracy = Sensitivity\ \times Analytical\ prevalence + Specificity\ \times (1 - Analytical\ Prevalence)$$

True Positives = SGTF in PCR and variant with ΔH69/V70
False Positives = SGTF in PCR and variant lacking ΔH69/V70
True Negatives = Triple positive in PCR and variant lacking ΔH69/V70
False Negatives = Triple positive in PCR and variant with ΔH69/V70
Analytical prevalence = Ratio of positive cases (True Positives + False Negatives) among all cases

Confidence intervals for sensitivity, specificity and accuracy are "exact" Clopper-Pearson confidence intervals.

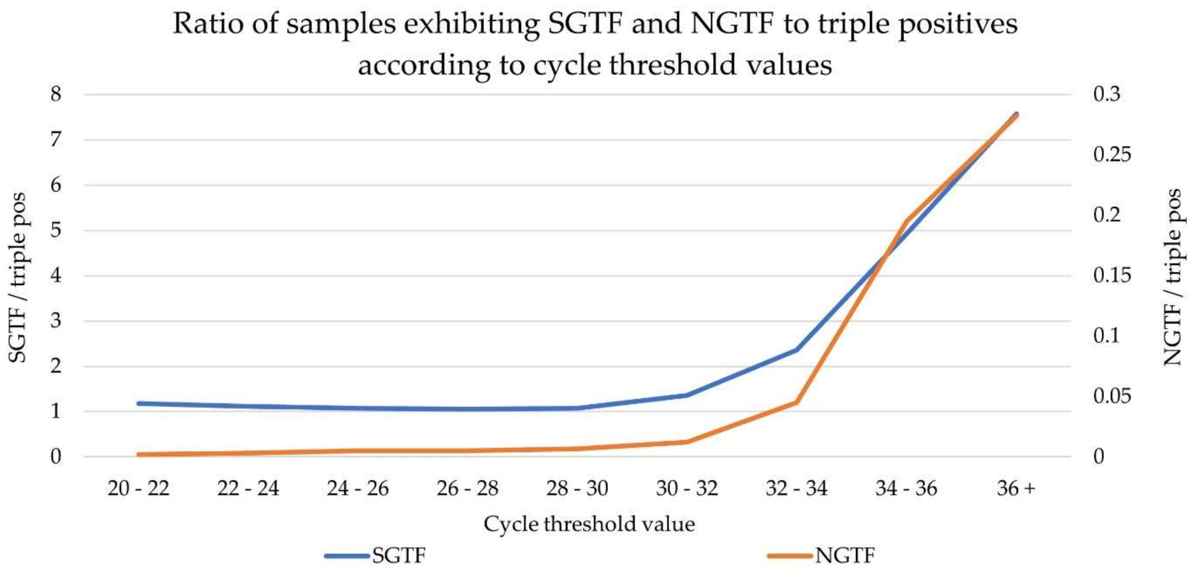

Figure S1. The ratio of samples presenting with S-gene target failure (SGTF) or N-gene target failure (NGTF) in relation to triple-positive samples. An increasing number of samples exhibiting SGTF or NGTF is observed when cycle threshold (CT) values for the ORF1ab target gene are greater than 30, as illustrated by the rising slope.

A decrease in median CT values of samples presenting with SGTF from approximately 30 to 20 was observed during the emergence of the alpha variant (Figure S2). Median CT values gradually decreased

until 50% SGTF was reached and then stayed constant during the period of dominance. This effect was reversed with the introduction of the delta variant where median CT values of SGTF positive samples increased again to 30+ values. The same effect, although not as substantial due to shorter transition periods, was observed during the change of delta to BA.1 (mean CT values decreased), BA.1 to BA.2 (mean CT values increased) and BA.2 to BA.5 (mean CT values decreased). These changes reflect the respective variants expansion in the population and the switch from nonspecific SGTF in low viral load samples to specific SGTF in high viral load samples.

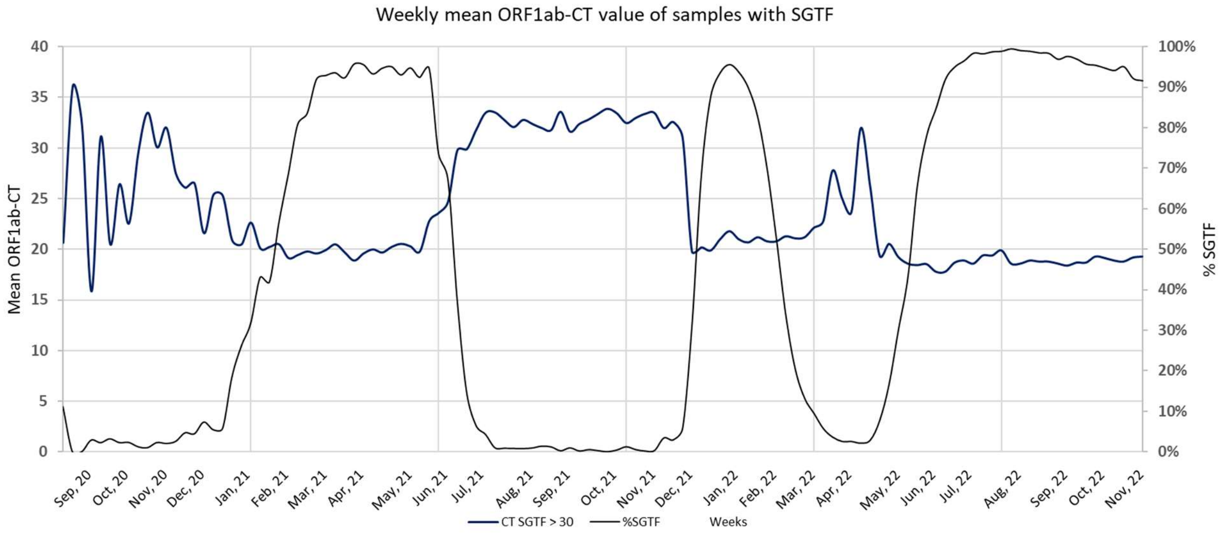

Figure S2. Weekly mean ORF1AB C_t-value of SGTF positive samples. The frequency of SGTF is plotted to compare to the emergence of new dominant variants. Depicted is the change from unspecific SGTF with C_t-value ~30 in low viral load samples to specific SGTF with C_t-value ~20 in high viral load samples during the expansion of new SGTF positive variants.

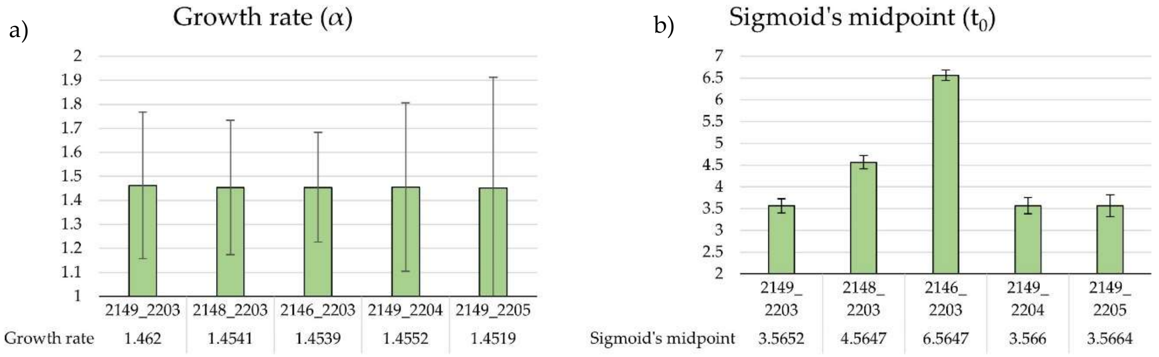

Figure S3. Sensitivity analysis for growth rate and sigmoid's midpoint depending on initialization of logistic fit to the dataset. The growth rate does not change significantly when the initialization point is moved in one-week intervals (a), while Sigmoid's midpoint changes accordingly (b). Depicted is the logistic fit for the Omicron BA.1 dataset, as this dataset is the least robust with the least datapoints due to its short emergence period.

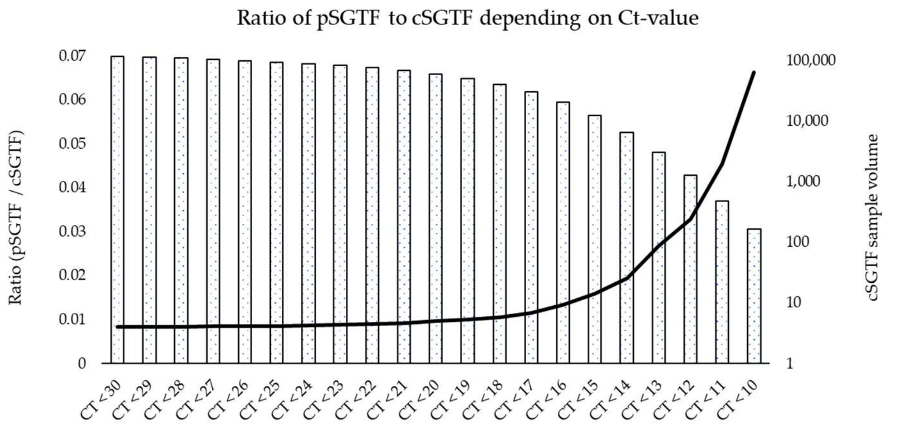

Figure S4. The occurrence of partial S-gene target failure (pSGTF) is dependent on the cycle-threshold (Ct) value. Depicted is the ratio of pSGTF (triple positives with S-gene shift > 4) to complete SGTF (cSGTF) according to Ct-values within our dataset. The presentation of pSGTF gets more frequent with decreasing Ct-value / increasing viral load in the sample, suggesting infrequent mismatch annealing if high numbers of S-gene amplicons are in the reaction mix.

Table S1. B.1.1.7* (Alpha) specific sensitivity and specificity.

VOC determination (Alpha)						
	TaqPath	alpha	Non-alpha	Total		
Total	SGTF positive	3615	13	3628	Sensitivity [95% CI]	98.21 [97.72, 98.61]
	SGTF negative	66	1658	1724	Specificity [95% CI]	99.22 [98.67, 99.59]
	Total	3681	1671	5352	Accuracy [95% CI]	98.52 [98.16, 98.83]
Total > 3.8	SGTF positive	3653	17	3670	Sensitivity [95% CI]	99.24 [98.90, 99.49]
	SGTF negative	28	1654	1682	Specificity [95% CI]	98.98 [98.38, 99.41]
	Total	3681	1671	5352	Accuracy [95% CI]	99.16 [98.88, 99.39]
WGS	SGTF positive	337	12	349	Sensitivity [95% CI]	97.68 [95.48, 98.99]
	SGTF negative	8	182	190	Specificity [95% CI]	93.81 [89.44, 96.76]
	Total	345	194	539	Accuracy [95% CI]	96.29 [94.33, 97.72]
WGS > 3.8	SGTF positive	342	13	355	Sensitivity [95% CI]	99.13 [97.48, 99.82]
	SGTF negative	3	181	184	Specificity [95% CI]	93.30 [88.81, 96.38]
	Total	345	194	539	Accuracy [95% CI]	97.03 [95.22, 98.29]
SNP	SGTF positive	3278	1	3279	Sensitivity [95% CI]	98.35 [97.86, 98.75]
	SGTF negative	55	1451	1506	Specificity [95% CI]	99.93 [99.62, 100.00]
	Total	3333	1452	4785	Accuracy [95% CI]	98.83 [98.48, 99.11]
SNP >3.8	SGTF positive	3308	4	3312	Sensitivity [95% CI]	99.25 [98.89, 99.51]
	SGTF negative	25	1448	1473	Specificity [95% CI]	99.72 [99.30, 99.92]
	Total	3333	1452	4785	Accuracy [95% CI]	99.39 [99.13, 99.59]

Table S2. B.1.617.2* (Delta) specific sensitivity and specificity.

VOC determination (Delta)							
	TaqPath	delta	Non-delta	Total			
Total	SGTF positive	0	10	10	Sensitivity [95% CI]	100.00%	99.46% to 100.00%
	SGTF negative	679	1	680	Specificity [95% CI]	90.91%	58.72% to 99.77%
	Total	679	11	690	Accuracy [95% CI]	99.86%	99.20% to 100.00%
Total > 3.8	SGTF positive	2	11	13	Sensitivity [95% CI]	99.71%	98.94% to 99.96%
	SGTF negative	677	0	677	Specificity [95% CI]	100.00%	71.51% to 100.00%
	Total	679	11	690	Accuracy [95% CI]	99.71%	98.96% to 99.96%
WGS	SGTF positive	0	10	10	Sensitivity [95% CI]	100.00%	98.46% to 100.00%
	SGTF negative	238	1	239	Specificity [95% CI]	90.91%	58.72% to 99.77%
	Total	238	11	249	Accuracy [95% CI]	99.60%	97.78% to 99.99%
WGS > 3.8	SGTF positive	1	11	12	Sensitivity [95% CI]	99.58%	97.68% to 99.99%
	SGTF negative	237	0	237	Specificity [95% CI]	100.00%	71.51% to 100.00%
	Total	238	11	249	Accuracy [95% CI]	99.60%	97.78% to 99.99%
SNP	SGTF positive	0	0	0	Sensitivity [95% CI]	100.00%	99.17% to 100.00%
	SGTF negative	441	0	441	Specificity [95% CI]	-	
	Total	441	0	441	Accuracy [95% CI]	-	
SNP >3.8	SGTF positive	1	0	1	Sensitivity [95% CI]	99.77%	98.74% to 99.99%
	SGTF negative	440	0	440	Specificity [95% CI]	-	
	Total	441	0	441	Accuracy [95% CI]	-	

Table S3. B.1.1.529.1* (BA.1) specific sensitivity and specificity.

VOC determination (BA.1)							
	TaqPath	BA.1	Non-BA.1	Total			
Total	SGTF positive	2994	5	2999	Sensitivity [95% CI]	93.94%	93.06% to 94.75%
	SGTF negative	193	1230	1423	Specificity [95% CI]	95.87%	94.63% to 96.89%
	Total	3187	1283	4422	Accuracy [95% CI]	94.50%	93.79% to 95.15%
Total > 3.8	SGTF positive	3171	9	3180	Sensitivity [95% CI]	99.50%	99.19% to 99.71%
	SGTF negative	16	1226	1242	Specificity [95% CI]	99.27%	98.62% to 99.67%
	Total	3187	1235	4422	Accuracy [95% CI]	99.43%	99.17% to 99.63%
WGS	SGTF positive	1092	5	1097	Sensitivity [95% CI]	100.00%	99.66% to 100.00%
	SGTF negative	0	285	285	Specificity [95% CI]	98.28%	96.02% to 99.44%
	Total	1092	290	1382	Accuracy [95% CI]	99.64%	99.16% to 99.88%
WGS > 3.8	SGTF positive	1092	6	1098	Sensitivity [95% CI]	100.00%	99.66% to 100.00%
	SGTF negative	0	284	284	Specificity [95% CI]	97.93%	95.55% to 99.24%
	Total	1092	290	1382	Accuracy [95% CI]	99.57%	99.06% to 99.84%
SNP	SGTF positive	1902	0	1902	Sensitivity [95% CI]	90.79%	89.47% to 91.99%
	SGTF negative	193	945	1138	Specificity [95% CI]	100.00%	99.61% to 100.00%
	Total	2095	945	3040	Accuracy [95% CI]	93.65%	92.73% to 94.49%
SNP >3.8	SGTF positive	2079	3	2082	Sensitivity [95% CI]	99.24%	98.76% to 99.56%
	SGTF negative	16	942	958	Specificity [95% CI]	99.68%	99.08% to 99.93%
	Total	2095	945	3040	Accuracy [95% CI]	99.37%	99.03% to 99.62%

Table S4. B.1.1.529.2* (BA.2) specific sensitivity and specificity.

VOC determination (BA.2)							
	TaqPath	BA.2	Non-BA.2	Total			
Total	SGTF positive	2	844	846	Sensitivity [95% CI]	99.91%	99.68% to 99.99%
	SGTF negative	2230	423	2653	Specificity [95% CI]	66.61%	63.94% to 69.21%
	Total	2232	1267	3499	Accuracy [95% CI]	87.85%	86.73% to 88.92%
Total > 3.8	SGTF positive	16	1134	1150	Sensitivity [95% CI]	99.28%	98.84% to 99.59%
	SGTF negative	2216	133	2349	Specificity [95% CI]	89.50%	87.68% to 91.14%
	Total	2232	1267	3499	Accuracy [95% CI]	95.74%	95.02% to 96.39%
WGS	SGTF positive	2	820	822	Sensitivity [95% CI]	99.74%	99.07% to 99.97%
	SGTF negative	770	11	781	Specificity [95% CI]	87.79%	85.52% to 89.83%
	Total	772	831	1603	Accuracy [95% CI]	93.20%	91.90% to 94.35%
WGS > 3.8	SGTF positive	4	825	829	Sensitivity [95% CI]	99.48%	98.68% to 99.86%
	SGTF negative	768	6	774	Specificity [95% CI]	99.28%	98.44% to 99.73%
	Total	772	831	1603	Accuracy [95% CI]	99.38%	98.86% to 99.70%
SNP	SGTF positive	0	24	24	Sensitivity [95% CI]	100.00%	99.75% to 100.00%
	SGTF negative	1460	412	1872	Specificity [95% CI]	5.50%	3.56% to 8.08%
	Total	1460	436	1896	Accuracy [95% CI]	78.27%	76.34% to 80.11%
SNP >3.8	SGTF positive	12	309	321	Sensitivity [95% CI]	99.18%	98.57% to 99.57%
	SGTF negative	1448	127	1575	Specificity [95% CI]	70.87%	66.36% to 75.10%
	Total	1460	436	1896	Accuracy [95% CI]	92.67%	91.40% to 93.80%

Table S5. B.1.1.529.4* / B.1.1.529.5* (BA.4 / BA.5) specific sensitivity and specificity.

VOC determination (BA.4 / BA.5)							
	TaqPath	BA.4 / BA.5	Non-BA.4 / BA.5	Total			
Total	SGTF positive	457	21	478	Sensitivity [95% CI]	99.35%	98.11% to 99.87%
	SGTF negative	3	340	343	Specificity [95% CI]	94.18%	91.25% to 96.36%
	Total	460	361	821	Accuracy [95% CI]	97.08%	95.68% to 98.12%
Total > 3.8	SGTF positive	460	21	481	Sensitivity [95% CI]	100.00%	99.20% to 100.00%
	SGTF negative	0	340	340	Specificity [95% CI]	94.18%	91.25% to 96.36%
	Total	460	361	821	Accuracy [95% CI]	97.44%	96.12% to 98.41%

Table S6. Sensitivity and specificity based on VOC determination with mutation specific PCR (SNP).

VOC determination (SNP)						
	TaqPath	SGTF VOC*	nSGTF VOC**	Total	* Variants – B.1.258, alpha, BA.1, BA.4/BA.5<br>** Variants – WT, delta, BA.2	
SGTF	SGTF positive	5202	9	5211	Sensitivity [95% CI]	90.2% [89.4%, 91.0%]
	SGTF negative	564	4257	4821	Specificity [95% CI]	99.8% [99.6%, 99.9%]
	Total	5766	4266	10032	Accuracy [95% CI]	94.3% [93.8%, 94.7%]
pSGTF > 3.8	SGTF positive	5610	33	5643	Sensitivity [95% CI]	97.3% [96.8%, 97.7%]
	SGTF negative	156	4233	4389	Specificity [95% CI]	99.2% [98.9%, 99.5%]
	Total	5766	4266	10032	Accuracy [95% CI]	98.1% [97.8%, 98.4%]

Table S7. Sensitivity and specificity based on VOC determination with whole-genome-sequencing (WGS).

VOC determination (WGS)						
	TaqPath	SGTF VOC*	nSGTF VOC**	Total	* Variants – B.1.258, alpha, BA.1, BA.4/BA.5<br>** Variants – WT, delta, BA.2	
SGTF	SGTF positive	3359	47	3406	Sensitivity [95% CI]	98.3% [97.8%, 98.7%]
	SGTF negative	57	4229	4286	Specificity [95% CI]	98.9% [98.5%, 99.2%]
	Total	3416	4276	7692	Accuracy [95% CI]	98.7% [98.4%, 98.9%]
pSGTF > 3.8	SGTF positive	3403	61	3464	Sensitivity [95% CI]	99.6% [99.4%, 99.8%]
	SGTF negative	13	4215	4228	Specificity [95% CI]	98.6% [98.2%, 98.9%]
	Total	3416	4276	7692	Accuracy [95% CI]	99.0% [98.8%, 99.2%]

Table S8. Dominant SARS-CoV-2 variants and the respective amount of samples identified in this study by either whole-genome sequencing (WGS) or mutation specific PCR (SNP).

**WHO label**	**Pango lineage**	**Total**	**WGS**	**SNP**	**% total**
WT	*WT*	1432	22	1410	8.1
alpha	*B.1.1.7**	3152	138	3014	17.8
delta	*B.1.617.2**	3837	2492	1345	21.6
Omicron BA.1	*B.1.1.529.1**	4129	1695	2434	23.3
Omicron BA.2	*B.1.1.529.2**	3017	1547	1470	17.0
Omicron BA.4	*B.1.1.529.4**	94	94	0	0.5
Omicron BA.5	*B.1.1.529.5**	1259	1259	0	7.1
Others		804	445	359	4.5
**Sum**		17724	7692	10032	
